# Symptomatic giant gastric hyperplastic polyp: a case report 

**Published:** 2021

**Authors:** Nicholas Dalkie, Andrew Lane, Bruce Lockett, Kamran Rostami

**Affiliations:** 1 *Department of Gastroenterology, Palmerston North Hospital, New Zealand *; 2 *Department of Pathology, Palmerston North Hospital, New Zealand *

## Abstract

Herein we describe a rare case of a 59-year-old male who was diagnosed with giant hyperplastic polyps after referring with symptoms of abdominal pain and vomiting, and associated red-flag symptoms of unintentional weight loss and early satiety.

## Introduction

 Gastroscopy is a common procedure indicated in many conditions that is performed in endoscopy units and allows for direct visualization of the upper gastrointestinal tract to the proximal duodenum (1). Incidental findings of gastric polyps, most frequently fundic gland polyps, are common when performing endoscopy for other indications and are usually considered inconsequential (2). Rarely, these polyps may cause bleeding or even outlet obstruction (3). The decision of whether or not to intervene with resection and the risk of malignant transformation and ongoing surveillance procedures are still topics of ongoing research. 

## Case report

A 59-year-old male was referred to the gastroenterology department by his general practitioner with symptoms of upper abdominal pain and vomiting associated with early satiety, poor appetite, and a 5-kg weight loss over a period of 6 weeks. During this time, he also noted occasional black stools, but these were intermittent enough that he was not duly concerned and did not present acutely to the hospital. The patient described abdominal pain in the left upper quadrant, consistently present as a dull ache with waves of sharp exacerbations. His vomiting was worse in the mornings, and he denied any hematemesis. 

The patient’s medical history was significant for ischemic heart disease, for which he takes aspirin; gout, with no recent need for steroids or NSAIDs; and previous atrial fibrillation, for which he was electrically cardioverted and did not require ongoing anticoagulation. 

His social history was significant for an excessive alcohol intake of 6-8 standard drinks per night, which he had been consuming for about 5 years. He is a non-smoker. There is no family history of any malignancy. 

Investigations revealed microcytic anemia with hemoglobin of 80 g/L and concurrent iron deficiency with a ferritin of 21 ug/L; the anemia has since normalized. His liver function tests were abnormal with an elevated ALP and GGT of 256 and 612, respectively. Ultrasonic examination demonstrated heterogeneity consistent with steatosis with a normal contour and no liver lesions. 

**Figure 1:**
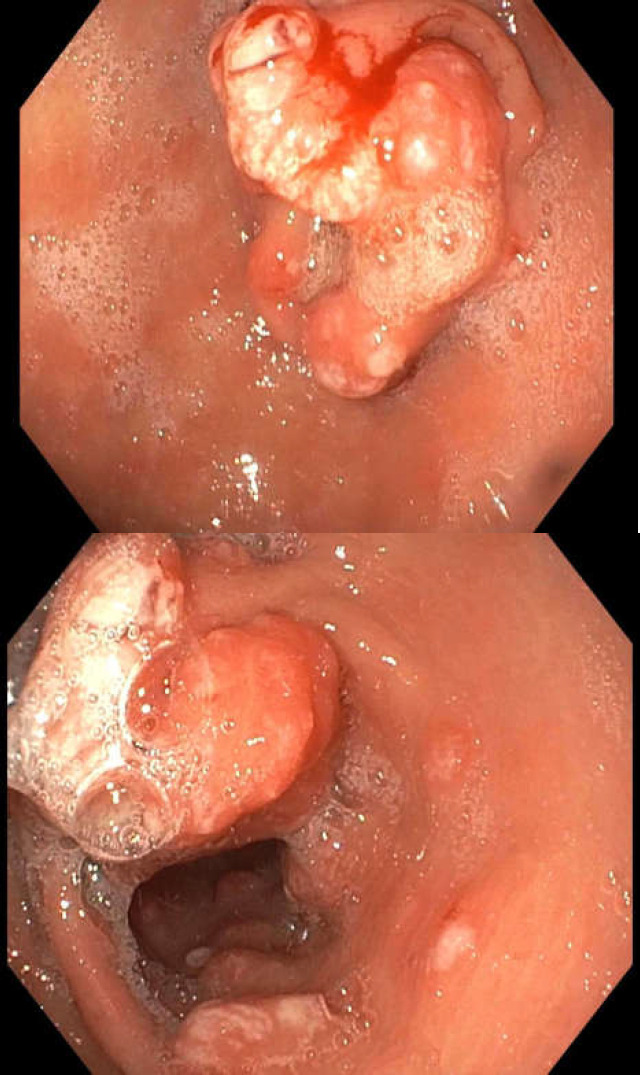
Endoscopic appearance of the gastric antral mass and satellite lesions of nodular mucosa

The patient came forward for a gastroscopy, the findings of which demonstrated a large pre-pyloric mass that was semi-circumferential, about 6-8 cm in length, and extended from the pylorus to involve most of one half of the gastric antrum. There was associated reflux esophagitis. Representative endoscopic pictures are shown in Figure 1. 

An urgent CT of his chest, abdomen, and pelvis demonstrated a gastric antral mass. Associated with this was minor, sub-centimeter, lymphadenopathy of the mediastinum, right hilum, and upper abdomen in the left gastric artery lymphatic basin. 

**Figure 2:**
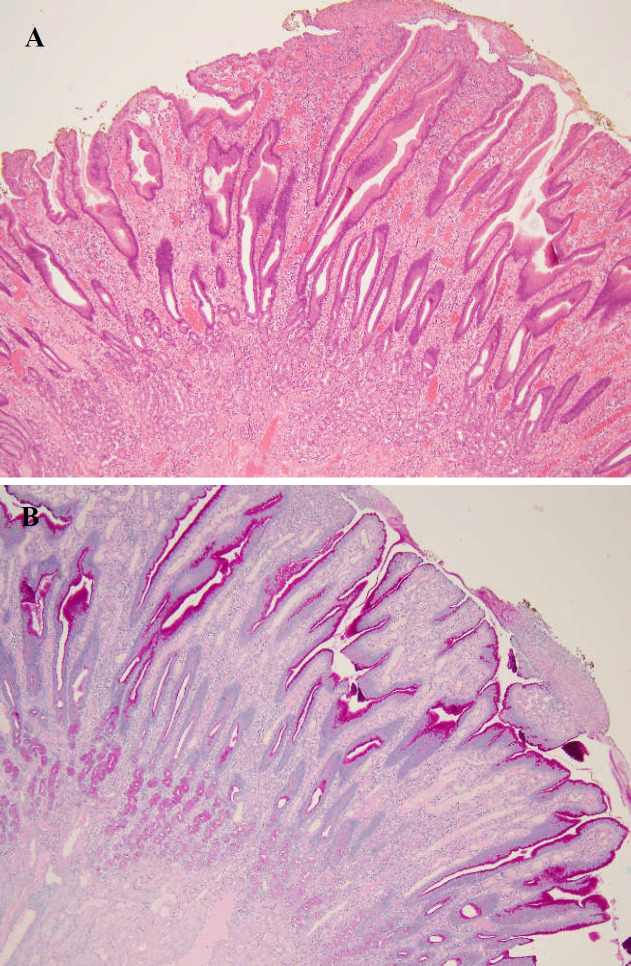
Histological sections of the gastric antral lesion with H&E stain (A) and ABPASD stain (B) Sections show inflamed, ulcerated gastric hyperplastic polyp. An Alcian blue/PASD stain for neutrophil and acid mucin shows no evidence of intestinal metaplasia or occult invasive carcinoma. Immunohistochemical techniques for Helicobacter pylori are negative

Initial biopsies of the lesion showed moderate non-specific mixed inflammatory changes with granulation tissue. The epithelium was reactive with atypia but no dysplasia. *Helicobacter pylori* was not present. Figure 2 demonstrates the histology of the lesion. 

Given the high suspicion of malignancy based on the endoscopic appearance, the radiology, and presumed non-representative biopsies, the patient underwent repeat gastroscopy. The repeat gastroscopy findings were that of prominent, nodular mucosa in the gastric antrum. The largest area of irregular mucosa again was noted to extend from the gastric antrum to the pylorus on the lesser curve aspect and was estimated endoscopically to be 6 cm in length and 3 cm in width. Further biopsies were taken.

## Discussion

Gastric hyperplastic polyps represent the second most common type of gastric polyps after fundic gland polyps (2). Endoscopically, they are seen to be either pedunculated or, more frequently, sessile and are usually antral in location with a typical size of less than 2 cm (3). Our patient presented with a larger polypoid mass that occurs in only 10% of these cases and are classified as giant gastric hyperplastic polyps (4). They appear to have a smooth but lobulated surface similar to what we can see in Figure 1, with the presence of erosions being a not uncommon feature (5). Narrow band imaging may be used to further assess the architecture of the vasculature, as this has been shown to correlate well with the subsequent histopathology (6). 

The majority of gastric hyperplastic polyps are found incidentally, as usually they are asymptomatic. However, giant gastric hyperplastic polyps may present with a variety of non-specific symptoms related to the upper gastrointestinal tract, including bleeding or anemia, dyspepsia, or even gastric outlet obstruction (5,6). In this respect, our patient presented with vomiting and abdominal pain that indicated a large antral mass, giving a similar presentation like cases reported in the current literature. Although presentations with massive bleeding are uncommon, it has been described in case reports (5). 

When examined histologically, gastric hyperplastic polyps consist of the foveolar cells that line the gastric mucosa; these cells branch and become stretched and abnormally edematous and vascular (4,7). Owing to their structure, they are readily exposed to trauma from ingested material and, as a result, are found to be chronically inflamed, which predisposes them to develop foci of atypia and dysplasia (4). Despite the development of dysplasia often found on biopsies of hyperplastic polyps, there is low malignant transformation of the polyps, although there is an increased risk of concurrent gastric malignancy when dysplasia is present (2). Even though the prevalence of dysplasia within polyps is estimated to be about 2%, perhaps higher in giant gastric hyperplastic polyps,^7^ it is suggested both adenomatous and hyperplastic polyps have malignant potential and are precursors of early gastric cancer. Polyp size has been regarded as an important factor with a correlation of the larger the lesion, the higher the chance for malignant transformation. Based on this, our patient with a polyp of such a large size would be at higher risk of developing dysplasia and of subsequent malignant transformation. Therefore, the decision was made to proceed to polypectomy with a follow-up surveillance gastroscopy at 1 year to assess.

The evidence to support our decision came from studies that recommend polypectomy on all polyps >2cm, as biopsy alone may miss foci of dysplasia and underestimate the malignant potential (3).

Association is noted with *Helicobacter pylori*, and our patient was negative for this. It is recommended to perform a biopsy of the surrounding flat mucosa to assess for infection and treat as appropriate, with around 80% of polyps regressing from treatment of *Helicobacter *alone (2). Biopsy of the surrounding mucosa also allows for assessment of any significant atrophic gastritis or intestinal metaplasia (7). These histological findings indicate the patient to be at increased risk of gastric malignancy; however the overall risk is low, and surveillance is not currently recommended in patients not deemed to be high risk (8). Despite this, a one-off follow-up surveillance gastroscopy is often performed at 1 year to re-biopsy and further assess (8).
